# Quality and readability of AI-generated information on bipolar disorder: a cross-sectional content analysis

**DOI:** 10.1186/s12888-026-08262-z

**Published:** 2026-06-10

**Authors:** Ibrahim Karakaya

**Affiliations:** Istanbul Gelisim University, Istanbul, Türkiye

**Keywords:** Bipolar disorder, Artificial intelligence, Large language models, Psychoeducation, Readability, Patient information, Digital mental health, EQIP

## Abstract

**Background:**

Bipolar disorder is a clinically sensitive and diagnostically complex condition in which unclear or incomplete psychoeducational information may contribute to misunderstanding of symptoms, delayed help-seeking, and unsafe interpretation of treatment options. Large language models are increasingly used as on-demand sources of mental health information, yet comparative evidence on the quality and readability of AI-generated information about bipolar disorder remains limited.

**Methods:**

This cross-sectional content analysis evaluated 180 responses generated by ChatGPT, Gemini, and DeepSeek to 20 bipolar disorder-related questions derived from Google Trends. Each question was asked in three independent new sessions for each model. Information quality was assessed using the 20-item EQIP instrument, and readability was evaluated using Flesch-Kincaid Grade Level, Flesch Reading Ease, and word count. To address the non-independence of repeated responses nested within prompts, a linear mixed-effects model was used with AI model and question category as fixed effects and question ID as a random intercept.

**Results:**

In the mixed-effects analysis, AI model significantly predicted EQIP scores. Compared with ChatGPT, Gemini and DeepSeek generated higher EQIP scores, with DeepSeek showing the largest estimated difference. Question category also contributed to information quality, although category-level pairwise comparisons did not remain significant after Bonferroni adjustment. Higher EQIP scores were moderately associated with longer responses and more favorable readability indices. Inter-rater analyses showed moderate absolute agreement for total EQIP scores and variable item-level agreement.

**Conclusions:**

Within the specific models, access conditions, prompts, date, and settings tested in this study, AI-generated bipolar disorder information differed across models in EQIP-rated quality and readability. These findings should be interpreted as content-quality findings rather than evidence of clinical accuracy, safety, or patient benefit. AI-generated psychoeducation should therefore be treated as a supplementary information source requiring expert review rather than a replacement for clinician-guided education.

**Supplementary Information:**

The online version contains supplementary material available at 10.1186/s12888-026-08262-z.

## Introduction

Bipolar disorder is not merely a mood disorder defined by episodes of mania, hypomania, and depression. It is a complex mental health condition that can have long-term effects on functioning, relationships, academic and professional life, daily decision-making, and quality of life. According to the World Health Organization, approximately 37 million people worldwide were living with bipolar disorder in 2021. Bipolar disorder is also associated with functional impairment, increased suicide risk, and common psychiatric comorbidities, including anxiety disorders and substance use disorders [1]. At the same time, access to treatment remains inadequate, misdiagnosis is common, and stigma complicates care processes [1].

Bipolar disorder represents a particularly relevant focus for evaluating AI-generated health information because it combines diagnostic complexity with substantial potential consequences of misinformation. Symptoms of bipolar depression may be confused with unipolar depression, hypomania may be minimized or misinterpreted as normal productivity, and manic symptoms may be recognized late by patients or families. In addition, patient-facing explanations of bipolar disorder often need to communicate risk-sensitive issues, including suicide risk, antidepressant-induced mood switching, medication discontinuation, substance use, pregnancy-related treatment considerations, and the need for specialist assessment. For these reasons, the quality and readability of AI-generated information may be especially consequential in bipolar disorder compared with more general mental health information.

Access to accurate, clear, and reliable information about bipolar disorder is therefore clinically important for early recognition, appropriate help-seeking, timely identification of symptoms, recurrence prevention, and long-term follow-up. Current guidelines indicate that the assessment and management of bipolar disorder should aim not only at symptom control but also at organizing care in a way that supports quality of life [2]. Psychoeducation has also been reported to reduce recurrence and hospitalization risk in bipolar disorder [3, 4]. Thus, the quality of patient-facing information is not a peripheral issue but a factor that may shape treatment engagement and outcomes.

Online resources can support the patient-physician encounter and, for some individuals, may become the main or initial source of information. However, the availability of large amounts of content does not ensure that such information is reliable, balanced, or understandable. This issue is especially important in mental health, where symptoms may affect insight, risk perception, and help-seeking. Recent work indicates that publicly available mental health information is often written above recommended patient reading levels and does not consistently implement plain-language principles [5–7].

The quality of online information on bipolar disorder has also long been debated. Studies have shown that internet resources on bipolar disorder vary in readability, transparency, and content quality, and that treatment-related information may differ substantially according to language, source type, and website structure [8, 9]. Therefore, the main problem is not simply whether information about bipolar disorder can be accessed, but whether the information accessed is accurate, balanced, understandable, and appropriate for patients and families.

Large language models such as ChatGPT, Gemini, and DeepSeek have changed how health information is produced and consumed. These systems generate direct, conversational answers rather than simply directing users to static web pages. Reviews of large language models in mental health describe potential applications in education, screening, classification, and clinical support, while also emphasizing limitations related to accuracy, reliability, interpretability, ethics, and overreliance [10, 11]. In clinically sensitive conditions such as bipolar disorder, these concerns are particularly important because incomplete or unsafe advice may influence help-seeking, medication perceptions, and risk recognition.

Recent studies suggest that large language models may improve the readability of patient education materials, although readability gains do not guarantee accuracy or safety [7, 12–14]. Much of the existing literature in mental health has focused on tasks such as detection, classification, or general clinical support. Comparative evidence on the quality and readability of AI-generated psychoeducational information specifically about bipolar disorder remains limited.

To address this gap, the present study compared responses generated by ChatGPT, Gemini, and DeepSeek to bipolar disorder-related questions derived from Google Trends. The study evaluated responses in terms of EQIP-rated information quality, readability, and response length. The aim was not to measure clinical outcomes, diagnostic accuracy, or patient safety directly. Rather, the study examined whether AI-generated information differed across models and predefined question categories and discussed the potential implications of these content-quality and readability findings for patient-facing psychoeducational information.

## Methods

### Study design and ethical considerations

This study was designed as a cross-sectional content analysis of AI-generated text outputs. It examined the information quality and readability of responses generated by large language models to questions related to bipolar disorder. Clinical relevance was not treated as a directly measured patient outcome; instead, the potential clinical implications of quality and readability findings were interpreted cautiously in relation to patient-facing psychoeducation. Because the study involved only AI-generated text outputs and did not include human participants, patient data, human tissue, animal subjects, or clinical records, ethics committee approval and informed consent were not required.

### Question generation

The most frequently searched keywords and questions related to bipolar disorder were identified using Google Trends. Searches were conducted to cover the 12-month period preceding the data collection date. The geographic filter was set to Worldwide, the search type was Web search, and the analyses were conducted using English search terms related to bipolar disorder. The related queries and related topics outputs were reviewed, semantically overlapping expressions were merged, and clinically relevant search expressions were transformed into patient-facing questions.

Google Trends was used to approximate real-world public information-seeking patterns rather than to define the full clinical risk profile of bipolar disorder. Therefore, the question set was intended to reflect commonly searched patient-facing topics, while recognizing that some misinformation-prone or high-risk domains may not appear among frequently searched queries.

The first evaluator initially transformed the Google Trends outputs into questions. A second evaluator independently reviewed the wording, relevance, and category assignment of the questions. The final version of the 20-question set was produced through discussion and agreement between the two evaluators before data collection. The final English question set used in the study is presented in Table 1.

**Table 1:** Final bipolar disorder question set used for AI prompting

Question ID	Question category	Question
Q01	Bipolar disorder	What is bipolar disorder?
Q02	Bipolar disorder	What is the difference between bipolar I and bipolar II disorder?
Q03	Bipolar disorder	What are the symptoms of bipolar disorder?
Q04	Bipolar disorder	What is the difference between borderline personality disorder and bipolar disorder?
Q05	Bipolar treatment	What is the treatment for bipolar disorder?
Q06	Bipolar treatment	Which medications are used in the treatment of bipolar disorder?
Q07	Bipolar treatment	What is lithium?
Q08	Manic episode	What is a manic episode?
Q09	Manic episode	What are the symptoms of a manic episode?
Q10	Manic episode	What triggers a manic episode?
Q11	Manic episode	How can a manic episode be stopped immediately?
Q12	Manic episode	What is hypomania?
Q13	Depressive episode	What is a depressive episode?
Q14	Depressive episode	How long does a depressive episode last?
Q15	Depressive episode	What is recurrent depressive disorder?
Q16	Mood stabilizers	What are mood stabilizers?
Q17	Mood stabilizers	What is an antipsychotic?
Q18	Mood stabilizers	What do mood stabilizers do?
Q19	Mood stabilizers	What are the side effects of mood stabilizers?
Q20	Mood stabilizers	Do mood stabilizers help with anxiety?

### Model access, settings, and reproducibility precautions

All AI-generated responses were collected on October 23, 2025. ChatGPT responses were generated through the ChatGPT web interface using the ChatGPT model available to the researcher under the active subscription/access status on the data collection date. Gemini responses were generated through the Google Gemini web interface under a Google AI Pro subscription using the Google AI Pro model available at the time. DeepSeek responses were generated through the DeepSeek web interface using the DeepSeek Pro interface available to the researcher at the time of data collection.

The exact backend build identifiers were not separately recorded because the study used consumer-facing web interfaces. Therefore, the findings should be interpreted as applying to the specific interfaces, access conditions, date, prompts, and settings used in this study. To reduce the influence of previous interactions, browser history and related data were cleared before data collection, and each question was entered in a new chat session. No additional files, external documents, custom instructions, custom GPTs, plugins, or separately activated web-browsing/retrieval-augmented features were used by the researcher.

### Data collection procedure

The 20 questions were posed to three artificial intelligence models while preserving the same question wording and order: ChatGPT, Gemini, and DeepSeek. Each question was entered into a separate new chat page. To assess response variability across repeated sessions, each question was asked in three independent new sessions for each model. This yielded 60 responses per model and a total of 180 unique AI-generated responses. All responses were saved as text files and evaluated for information quality and readability.

### Assessment of information quality and readability

Information quality was assessed using the 20-item short form of the Ensuring Quality Information for Patients (EQIP) instrument. EQIP is a validated instrument developed to assess the quality of written health information [24]. Each item was scored as yes, partly, no, or not applicable. A yes response was scored as 1 point, partly as 0.5 points, and no as 0 points. Items marked as not applicable were excluded from the denominator. For each response, the EQIP percentage score was calculated by dividing the total obtained score by the number of applicable items and multiplying by 100.

Because EQIP was originally developed for written patient information materials rather than conversational AI responses, item-level operationalization was defined before scoring. Some document-specific items, such as visual, layout, or accessibility features that could not be meaningfully assessed in plain text AI outputs, were handled as not applicable when appropriate. The full English operationalization table is provided separately as Supplementary Table S1.

Readability was assessed using Flesch-Kincaid Grade Level (FKGL) and Flesch Reading Ease (FRE). FKGL estimates the approximate education level required to understand a text, whereas FRE estimates reading ease, with higher scores indicating easier readability. Word count was also calculated for each response as an indicator of response length.

### Inter-rater agreement

EQIP scoring was performed independently by two raters, a psychiatrist and a clinical psychologist. Inter-rater agreement for total EQIP scores was evaluated using Spearman rank correlation, Pearson correlation, and the intraclass correlation coefficient ICC(2,1). Pearson and Spearman correlations were interpreted as indicators of association or ranking similarity, whereas ICC(2,1) was interpreted as absolute agreement. Weighted kappa coefficients were calculated to evaluate item-level agreement where sufficient score variability was available.

### Statistical analysis

Descriptive statistics were calculated for EQIP scores, word count, FKGL, and FRE by AI model. Because the 180 responses were not fully independent observations, the primary analysis used a linear mixed-effects model rather than treating all responses as unrelated observations. Total EQIP score was entered as the dependent variable. AI model and question category were included as fixed effects, and question ID was included as a random intercept to account for clustering of repeated responses within prompts. Post-hoc pairwise comparisons were conducted using model-estimated marginal differences with Bonferroni adjustment. Adjusted p-values are explicitly labeled as adjusted. Correlations between EQIP scores and readability metrics were reported with 95% confidence intervals. Effect sizes were reported for pairwise model and category comparisons.

## Results

A total of 180 unique responses generated by ChatGPT, Gemini, and DeepSeek across 20 bipolar disorder-related questions were included in the final analysis. The responses were evaluated according to information quality, readability metrics, and response length (Table 2).

**Table 2:** Descriptive statistics of EQIP and readability metrics by AI model

Model	EQIP Score M (SD)	Word Count M (SD)	FKGL M (SD)	FRE M (SD)
ChatGPT	54.69 (4.34)	199.58 (8.16)	13.07 (1.04)	31.32 (6.20)
Gemini	60.30 (4.35)	211.15 (11.20)	12.79 (1.10)	29.51 (5.67)
Deepseek	64.67 (6.79)	228.42 (24.12)	11.70 (1.21)	39.16 (7.30)
Total	59.89 (6.67)	213.05 (19.90)	12.53 (1.26)	33.33 (7.65)

Note. FKGL = Flesch-Kincaid Grade Level; FRE = Flesch Reading Ease. M = Mean; SD = Standard Deviation

The overall mean EQIP score was 59.89 (SD = 6.67), indicating moderate information quality. Descriptively, DeepSeek generated the highest mean EQIP score and the longest responses, and it also showed the lowest mean FKGL and highest mean FRE. However, model differences were interpreted primarily through the mixed-effects model presented in Table 3.

**Table 3:** Linear mixed-effects model predicting total EQIP scores

Fixed Effects	Estimate (β)	SE	95% CI	z	*p*
Intercept (ChatGPT, Bipolar Disorder)	51.94	1.50	[48.99, 54.88]	34.55	< 0.001*
**Model (Ref = ChatGPT)**					
Gemini	5.61	0.81	[4.02, 7.21]	6.91	< 0.001*
Deepseek	9.98	0.81	[8.39, 11.57]	12.28	< 0.001*
**Category (Ref = Bipolar Disorder)**					
Bipolar Treatment	4.52	2.18	[0.24, 8.80]	2.07	0.038*
Depressive Episode	5.59	2.18	[1.31, 9.86]	2.56	0.010*
Manic Episode	1.22	1.92	[–2.54, 4.97]	0.64	0.526
Mood Stabilizers	3.73	1.92	[–0.03, 7.49]	1.95	0.052
**Random Effects**	**Variance**				
Question ID (Intercept)	5.96				
Residual	19.80				

Note. Marginal R² = 0.447; Conditional R² = 0.575. CI = Confidence Interval.* p <.05

The linear mixed-effects model showed that AI model significantly predicted EQIP scores after accounting for clustering by question ID. Compared with ChatGPT, Gemini had higher EQIP scores (β = 5.61, *p* <.001), and DeepSeek had higher EQIP scores (β = 9.98, *p* <.001). Question category also contributed to information quality. Responses to depressive episode and bipolar treatment questions showed higher estimated EQIP scores relative to general bipolar disorder questions, whereas manic episode and mood stabilizer questions did not differ significantly from the reference category. Post-hoc pairwise comparisons between AI models are shown in Table 4.

**Table 4:** Post-hoc pairwise comparisons of AI models based on the linear mixed model (LMM)

Comparison Pair	Estimate (Δ)	SE	95% CI	Adjusted *p*	Cohen’s d
Deepseek vs. ChatGPT	9.98	0.81	[8.39, 11.57]	< 0.001*	2.24
Deepseek vs. Gemini	4.37	0.81	[2.78, 5.95]	< 0.001*	0.98
Gemini vs. ChatGPT	5.61	0.81	[4.02, 7.21]	< 0.001*	1.26

Note. *p*-values were adjusted using the Bonferroni correction method. Cohen’s *d* values indicate effect size magnitude.* p <.05

Post-hoc model comparisons indicated that all three models differed significantly from one another in EQIP-rated information quality. DeepSeek showed higher estimated EQIP scores than both ChatGPT and Gemini, and Gemini showed higher estimated EQIP scores than ChatGPT. These findings indicate model-level differences in written information quality within the tested dataset, but they do not establish clinical superiority or patient-use safety (Table 4). Post-hoc pairwise comparisons between question categories are shown in Table 5.

**Table 5:** Post-hoc pairwise comparisons of question categories based on the linear mixed model (LMM)

Comparison Pair	Estimate (Δ)	SE	95% CI	Adjusted *p*	Cohen’s d
Depressive Episode vs. Bipolar Disorder	5.59	2.18	[1.31, 9.86]	0.105	1.26
Depressive Episode vs. Manic Episode	4.37	2.08	[0.28, 8.46]	0.362	0.98
Bipolar Treatment vs. Bipolar Disorder	4.52	2.18	[0.24, 8.80]	0.384	1.02
Bipolar Treatment vs. Manic Episode	3.3	2.08	[–0.79, 7.39]	1.000	0.74
Mood Stabilizers vs. Bipolar Disorder	3.73	1.92	[–0.03, 7.49]	0.516	0.84
Mood Stabilizers vs. Manic Episode	2.51	1.80	[–1.03, 6.05]	1.000	0.56
Depressive Episode vs. Mood Stabilizers	1.86	2.08	[–2.23, 5.94]	1.000	0.42
Bipolar Treatment vs. Depressive Episode	–1.07	2.33	[–5.64, 3.50]	1.000	0.24
Bipolar Treatment vs. Mood Stabilizers	0.79	2.08	[–3.30, 4.88]	1.000	0.18
Manic Episode vs. Bipolar Disorder	1.22	1.92	[–2.54, 4.97]	1.000	0.27

Note. *p* values were adjusted for 10 multiple comparisons using the Bonferroni correction method. CI = Confidence Interval. *p <.05

After Bonferroni correction, no pairwise comparison between question categories remained statistically significant. Thus, although the fixed-effects model suggested some category-level differences relative to the reference category, the category findings should be interpreted more cautiously than the model-level differences (Table 5). Correlations between information quality and readability metrics are presented in Table 6.

**Table 6:** Correlations between information quality and readability metrics

Relationship	*r*	95% CI	*p*
EQIP Score vs. Word Count	0.39	[0.26, 0.51]	< 0.001*
EQIP Score vs. FKGL	–0.33	[–0.46, –0.20]	< 0.001*
EQIP Score vs. FRE	0.31	[0.17, 0.44]	< 0.001*
FKGL vs. FRE	–0.87	[–0.90, –0.83]	< 0.001*
Word Count vs. FKGL	–0.33	[–0.46, –0.20]	< 0.001*
Word Count vs. FRE	0.35	[0.22, 0.48]	< 0.001*

Note. *N* = 180. Confidence intervals were computed using Fisher’s *z*-transformation. *p <.05

Higher EQIP scores were moderately associated with longer responses, lower FKGL scores, and higher FRE scores. These findings suggest that, in this dataset, responses with higher information quality tended to be more comprehensive and more readable. However, readability formulas do not evaluate factual accuracy, clinical safety, emotional tone, cultural appropriateness, or actionability (Table 6). Inter-rater agreement metrics for total EQIP scores are presented in Table 7.

**Table 7:** Inter-rater agreement metrics for total EQIP scores

Metric	Value	*p*-value
Spearman’s Rank Correlation (ρ)	0.55	< 0.001*
Pearson Correlation (r)	0.58	< 0.001*
Intraclass Correlation (ICC 2,1)	0.57	< 0.001*

*p <.05

The inter-rater analyses indicated that the two raters tended to evaluate responses similarly in relative terms, but absolute agreement was moderate rather than strong. This distinction is important because correlation does not prove agreement in absolute scoring (Table 7). Item-level weighted kappa results are shown in Table 8.

**Table 8:** Item-level inter-rater agreement (Weighted Kappa)

EQIP Item	Kappa (κ)	Agreement Level
Q07	1.00	Perfect
Q15	0.89	Almost Perfect
Q04	0.84	Almost Perfect
Q20	0.79	Substantial
Q16	0.62	Substantial
Q02	0.58	Moderate
Q10	0.46	Moderate
Q17	0.44	Moderate
Others	0.22–0.26	Fair
Q03, Q06	0.00	No Agreement

Note. Interpretation of weighted kappa coefficients was based on conventional agreement benchmarks. Items Q05, Q08, Q09, Q11, Q12, and Q13 are not included in the table as they had zero variance (i.e., uniform scoring or “Not Applicable” across all responses), which precludes the calculation of kappa statistics. *p* <.05

Item-level agreement was heterogeneous. Some items showed substantial to perfect agreement, whereas other items showed only fair or no agreement. This pattern indicates that several EQIP criteria were more vulnerable to subjective interpretation when applied to conversational AI outputs, particularly items requiring judgments about the emphasis of key messages, benefit framing, and structural organization (Table 8).

## Discussion

The findings of this study show that AI-generated responses related to bipolar disorder differed across models in EQIP-rated information quality, readability, and response length. Although the generally moderate EQIP scores suggest that large language models have some capacity to generate structured psychoeducational information in mental health, the observed differences between models indicate that this capacity is not equivalent across systems. DeepSeek received the highest EQIP scores, followed by Gemini, whereas ChatGPT showed the lowest mean score. However, these differences should be interpreted as variation in measured written information quality rather than evidence that any model is clinically superior, safer, or preferable for patient use. This interpretation is consistent with comparative studies in other medical fields showing that model outputs may differ in completeness, readability, reliability, and usefulness [13, 14].

The focus on bipolar disorder is especially important in the context of AI-generated health information because this condition is particularly vulnerable to misunderstanding and clinically consequential misinformation. Bipolar depression may be mistaken for unipolar depression, hypomania may be minimized or interpreted as normal productivity, manic symptoms may be misunderstood as personality or behavioral problems, and bipolar disorder may be confused with borderline personality disorder. In addition, incomplete information about antidepressant use, medication discontinuation, substance use, suicide risk, mixed features, pregnancy, or emergency care may lead users to underestimate the need for specialist assessment. Therefore, bipolar disorder represents a high-priority condition for evaluating AI-generated psychoeducational content, not only because of its burden, but also because misinterpretation of symptoms and treatment information can have serious clinical implications.

An important point is that high-quality patient information does not merely consist of presenting a large amount of content. Instruments such as EQIP evaluate the scope, organization, balance, and user orientation of health information. In this context, DeepSeek’s higher scores may be related not only to providing more information, but also to presenting that information in a clearer and more organized way. In contrast, ChatGPT’s lower scores suggest that, in some responses, the model provided less complete or less user-oriented information. These differences may be related to model-specific response style, training data, safety behavior, and summarization tendencies. This interpretation is consistent with previous work reporting that responses to the same health question may vary across models in scope, clarity, and usefulness [13, 14].

The findings also suggest that the quality of AI-generated information may vary across predefined question categories rather than across an undefined question type or presentation format. In this study, the categories were specified before analysis as bipolar disorder, bipolar treatment, manic episode, depressive episode, and mood stabilizers. Questions about depressive episodes showed higher estimated EQIP scores than general bipolar disorder questions in the fixed-effects model, although category-level pairwise comparisons did not remain statistically significant after Bonferroni correction. Thus, the category findings should be interpreted cautiously. Still, the descriptive pattern is clinically meaningful because bipolar disorder is a multidimensional condition: broad questions require the integration of symptoms, differential diagnosis, course, risks, treatment, and long-term management, whereas narrower questions may be easier for models to structure clearly.

The relatively higher quality scores for depressive episode questions may also reflect the greater visibility of depressive symptoms in both clinical literature and public mental health discourse. Depression is often described more frequently and in greater detail than mania or hypomania in publicly available information. In contrast, manic and hypomanic symptoms may be presented superficially, misunderstood, or normalized. Evaluations of internet-based mental health information show that online content can be useful but may also reinforce misunderstandings [15]. Therefore, it is clinically important that models did not show equally strong performance across all bipolar-related content areas. Inadequate or overly general information about mania may delay appropriate help-seeking and prevent users from recognizing the seriousness of symptoms [15].

The use of Google Trends-derived questions was valuable because it allowed the study to focus on topics that reflect common public search behavior. However, frequently searched questions should not be assumed to capture all clinically high-risk or misinformation-prone aspects of bipolar disorder. For example, users may search general questions such as what bipolar disorder is or how a manic episode can be stopped, but Google Trends may not fully capture more specific safety-critical concerns such as antidepressant-induced mania, mixed states, suicide risk, pregnancy-related medication decisions, or abrupt medication discontinuation. Therefore, the question set should be understood as reflecting common public information needs rather than a complete map of bipolar disorder misinformation risks.

One of the strengths of this study is that it assessed information quality and readability together. DeepSeek not only produced higher EQIP scores, but also showed the most favorable descriptive readability profile, with the lowest Flesch-Kincaid Grade Level and the highest Flesch Reading Ease score. In contrast, responses generated by ChatGPT and Gemini required higher literacy levels. This is relevant for patient-facing information because health information is useful only if users can understand it. The recommended reading level for health materials is often described as approximately the sixth- to eighth-grade range, yet many real-world patient materials exceed this level [16, 17]. Online mental health information has similarly been reported to require high reading levels, which may limit equitable access to information [18].

The readability findings should nevertheless be interpreted carefully. Although higher EQIP scores were associated with lower FKGL and higher FRE scores, readability formulas cannot determine whether the content is clinically accurate, safe, emotionally appropriate, culturally sensitive, or actionable. This distinction directly addresses the clinical interpretation of the present findings: readability and EQIP-rated structure may indicate that a response is easier to process, but they do not prove that it is clinically correct or safe. Recent studies suggest that large language models may improve the readability of patient education materials, but they also emphasize the need for human oversight regarding accuracy, balance, and reliability [7, 19, 20].

The moderate positive relationship between response length and information quality complements this interpretation. In a complex topic such as bipolar disorder, higher-quality responses may require more explanatory detail because adequate psychoeducation should address symptoms, risks, treatment options, and help-seeking. However, length alone should not be equated with quality. Effective educational materials must be comprehensive, understandable, well structured, and responsive to user needs [13, 21]. In this study, the association between higher EQIP scores and more favorable readability suggests that comprehensive responses can also be relatively accessible when information is organized clearly. This is consistent with evidence that well-constructed AI-generated medical explanations may improve understanding without necessarily oversimplifying the content [7, 19, 20].

Nevertheless, the readability findings are not clinically sufficient on their own. Although DeepSeek appeared more readable than the other models, all models remained above ideal patient reading-level recommendations [16, 17]. This suggests that AI-generated bipolar disorder responses may still be difficult for many patients or family members to use directly as psychoeducational material. This issue is particularly important in bipolar disorder because insight, attention, risk perception, and decision-making may fluctuate across mood states. Information must therefore be not only readable in a formula-based sense, but also safe, clear, appropriately framed, and clinically reviewed. The present findings indicate that LLMs may approach some readability goals, but they do not yet meet the standards required for independent patient education [7, 19].

The inter-rater findings also require a cautious interpretation. Pearson and Spearman correlations indicate that the two raters tended to rank responses similarly, but these coefficients do not establish absolute agreement. The ICC value remained moderate, indicating that some dimensions of EQIP scoring were open to interpretation. Item-level variability further suggests that applying EQIP to conversational AI outputs is not entirely mechanical. Items involving key-message emphasis, realistic benefit framing, organization, and practical usefulness may require clinical judgment. This does not invalidate the findings, but it indicates that future studies should use detailed scoring manuals, calibration procedures, and possibly consensus scoring for difficult items.

The phrase clinical relevance should therefore be understood in a limited and interpretive sense in this study. The study did not directly measure patient outcomes such as symptom recognition, help-seeking behavior, treatment adherence, understanding, or real-world usefulness. Instead, it evaluated information quality and readability as features that may have implications for patient-facing psychoeducation. For this reason, the findings support cautious discussion of potential clinical implications, but not claims about direct clinical benefit or harm. This distinction is essential because a response can be well organized and readable while still omitting critical warnings or providing incomplete advice.

This study is important because generative AI has changed the format of digital mental health information. Previous studies primarily examined the quality of websites related to bipolar disorder and the limitations of online content [8, 9]. With large language models, users no longer encounter only static web pages; they receive dynamic, fluent, and personalized-seeming answers in real time. Such responses may appear authoritative even when they have not been clinically verified. This transformation indicates that AI-generated mental health information should be evaluated as a distinct form of digital health communication [13, 22, 23].

For this reason, the practical importance of these findings should not be overlooked, but it should be framed carefully. AI-based tools may increasingly become a first point of reference for individuals seeking information about bipolar disorder. Even if they are not formally part of healthcare delivery, they may influence how people understand symptoms, treatment options, and the need for professional support. Current digital psychiatry literature recognizes the potential of these technologies to facilitate access to information while also emphasizing risks related to trust, ethics, misinformation, overreliance, and lack of human oversight [22, 23]. In bipolar disorder, where misinterpretation can be clinically consequential and stigma remains a barrier to care, AI-assisted information generation is not merely a technical issue but a patient-safety and psychoeducation issue [8].

Overall, the findings suggest that large language models can generate moderately structured and readable information about bipolar disorder, but their outputs remain incomplete as standalone patient education resources. The safest interpretation is that these tools may support information seeking when used alongside clinician guidance and expert-reviewed materials. They should not be treated as substitutes for clinical assessment, diagnosis, treatment planning, crisis intervention, or medication advice. Future research should examine different languages, user groups, and clinical scenarios, and should combine quality and readability metrics with expert accuracy ratings, safety checklists, and patient or caregiver evaluations.

### Limitations

This study has several limitations. First, only three AI models were evaluated, and the results cannot be generalized to all large language models or future versions of the same models. Second, model outputs are time-sensitive; updates to model architecture, safety filters, retrieval functions, or interface defaults may change response quality. Therefore, the findings are limited to the specific models, interfaces, access conditions, prompts, date, and settings used in data collection.

Third, Google Trends was used to identify commonly searched bipolar disorder-related topics, but this approach may not capture all areas that are clinically high-risk or especially prone to misinformation. For example, queries about suicide, antidepressant-induced mania, mixed features, pregnancy, medication discontinuation, or substance use may be clinically crucial even if they are not among the most frequent search outputs. Thus, the question set reflects public search interest rather than a comprehensive safety checklist for bipolar disorder psychoeducation.

Fourth, although the revised mixed-effects model accounted for clustering by question ID, the repeated-session structure and small number of prompts per category limit the complexity of the random-effects structure that could be modeled. Fifth, EQIP was originally developed for written patient information materials and may not fully fit conversational AI outputs. Some items were less applicable to plain text responses, and not-applicable ratings were therefore handled item by item.

Sixth, the study did not directly measure clinical relevance as a patient outcome. Symptom recognition, help-seeking behavior, treatment engagement, patient understanding, and clinician-rated usefulness were not assessed. Consequently, clinical implications should be interpreted as limited inferences from information quality and readability rather than as evidence of actual patient benefit.

Finally, accuracy, safety, stigma, appropriateness for high-risk situations, and patient outcomes were not directly assessed. This is a major conceptual limitation. A response can be readable and well organized while still omitting clinically crucial warnings or providing incomplete advice. Future studies should include expert clinical accuracy ratings, bipolar disorder-specific safety checklists, DISCERN [25] or PEMAT [26] assessments, and patient or caregiver evaluations.

## Conclusion

Within the tested models, access conditions, date, prompts, and settings, AI-generated information on bipolar disorder differed across models in EQIP-rated quality and readability. DeepSeek showed the highest EQIP scores and the most favorable readability profile, Gemini showed intermediate performance, and ChatGPT showed comparatively lower scores. These findings indicate differences in measured information quality, not evidence that any model is clinically preferable or safe for patient use. EQIP and readability metrics should be interpreted as indicators of written information quality rather than as evidence of factual accuracy, clinical safety, or patient benefit. AI-generated bipolar disorder information should therefore be treated as supplementary and should be reviewed by qualified professionals before use in patient-facing psychoeducation.

## Supplementary Information

Below is the link to the electronic supplementary material.

Supplementary Material 1

## Data Availability

The datasets generated and/or analyzed during the current study are not publicly available because they consist of AI-generated text responses and evaluator scoring files prepared for the present content analysis, but they are available from the corresponding author on reasonable request.

## Declarations

### Author information

I.K. is affiliated with Istanbul Gelisim University, Istanbul, Türkiye.

## Abbreviations

AI: Artificial intelligence
EQIP: Ensuring Quality Information for Patients
FKGL: Flesch-Kincaid Grade Level
FRE: Flesch Reading Ease
ICC: Intraclass Correlation Coefficient
LLM: Large language model
LMM: Linear mixed-effects model
PEMAT: Patient Education Materials Assessment Tool
