# Supplementary material for: Quality and readability of AI-generated information on bipolar disorder: a cross-sectional content analysis
Source: BMC Psychiatry. 2026 Jun 10;26:630. doi: 10.1186/s12888-026-08262-z (PMC13479570; doi:10.1186/s12888-026-08262-z)
Supplement: Supplementary file 1 [file 12888_2026_8262_MOESM1_ESM.docx]

**Supplementary Table S1. Operationalization of EQIP Items for AI-Generated Bipolar Disorder Responses**

The table below presents the English item labels and operational questions used to apply the 20-item EQIP framework to AI-generated plain-text responses. Items that were not applicable to a given response were excluded from the denominator when calculating the EQIP percentage score.

**Supplementary Table S1. EQIP item content and operationalization**

| **EQIP item** | **Domain** | **Operationalized item content** |
| --- | --- | --- |
| Q1 | Purpose and target audience | Is the purpose of the material clearly stated? |
| Q2 | Purpose and target audience | Is the target audience clearly specified? |
| Q3 | Purpose and target audience | Are key messages emphasized in a way that supports decision-making? |
| Q4 | Content accuracy and scope | Is the information current, evidence-based, and referenced? |
| Q5 | Content accuracy and scope | Are medical terms explained simply when needed? |
| Q6 | Content accuracy and scope | Are benefits described realistically and clearly? |
| Q7 | Content accuracy and scope | Are risks or side effects presented in a balanced and understandable way? |
| Q8 | Content accuracy and scope | Are alternative options mentioned? |
| Q9 | Content accuracy and scope | Are warning signs and indications for seeking medical help provided? |
| Q10 | Actionability and guidance | Are concrete step-by-step actions provided? |
| Q11 | Actionability and guidance | Are numerical data presented clearly when relevant? |
| Q12 | Actionability and guidance | Are uncertainties or limits of evidence stated honestly? |
| Q13 | Actionability and guidance | Does the material encourage shared decision-making and asking questions? |
| Q14 | Language, style, and presentation | Is the language plain, with short sentences and active wording? |
| Q15 | Language, style, and presentation | Is the readability level appropriate for the target audience? |
| Q16 | Language, style, and presentation | Do visuals or tables support the text and include explanatory headings/captions? |
| Q17 | Language, style, and presentation | Is the layout well organized with headings, bullet points, and spacing? |
| Q18 | Accessibility and inclusiveness | Is inclusive, non-judgmental, and non-stigmatizing language used? |
| Q19 | Accessibility and inclusiveness | Are accessibility features considered, such as font size, contrast, or alternative text when applicable? |
| Q20 | Accessibility and inclusiveness | Are contact routes or reliable additional resources provided? |

*Note. NA ratings were applied item by item when the criterion could not be meaningfully assessed for a plain-text AI response or when the prompt did not require the relevant content.*
